# Toll-like receptor 4 is a master regulator for colorectal cancer growth under high-fat diet by programming cancer metabolism

**DOI:** 10.1038/s41419-021-04076-x

**Published:** 2021-08-12

**Authors:** Xianjing Hu, Sarwat Fatima, Minting Chen, Keyang Xu, Chunhua Huang, Rui-Hong Gong, Tao Su, Hoi Leong Xavier Wong, Zhaoxiang Bian, Hiu Yee Kwan

**Affiliations:** 1 Centre for Cancer and Inflammation Research, School of Chinese Medicine, Hong Kong Baptist University, Hong Kong, China; 2 International Institute for Translational Chinese Medicine, Guangzhou University of Chinese Medicine, Guangzhou, Guangdong, 510006 China

**Keywords:** Cancer metabolism, Colorectal cancer

## Abstract

Although high-fat diet (HFD) has been implicated in the development of colorectal cancer (CRC), the critical signaling molecule that mediates the cancer growth is not well-defined. Identifying the master regulator that controls CRC growth under HFD can facilitate the development of effective therapeutics for the cancer treatment. In this study, the global lipidomics and RNA sequencing data show that, in the tumor tissues of CRC-bearing mouse models, HFD not only increases tumor weight, but also the palmitic acid level and TLR4 expression, which are reduced when HFD is replaced by control diet. These concomitant changes suggest the roles of palmitic acid and TLR4 in CRC growth. Subsequent studies show that palmitic acid regulates TLR4 expression in PU.1-dependent manner. Knockdown of PU.1 or mutations of PU.1-binding site on TLR4 promoter abolish the palmitic acid-increased TLR4 expression. The role of palmitic acid/PU.1/TLR4 axis in CRC growth is further examined in cell model and animal models that are fed either HFD or palmitic acid-rich diet. More importantly, iTRAQ proteomics data show that knockdown of TLR4 changes the metabolic enzyme profiles in the tumor tissues, which completely abolish the HFD-enhanced ATP production and cancer growth. Our data clearly demonstrate that TLR4 is a master regulator for CRC growth under HFD by programming cancer metabolism.

## Introduction

Studies demonstrate that inflammation, changes in gut microbiota and release of adipokines under obesity conditions are the players that regulate colorectal cancer (CRC) growth [1–3]. Nevertheless, the master regulator that controls CRC growth under high-fat dietary intervention has not been well-defined [4].

Toll-like receptor 4 (TLR4) is a pattern recognition receptor. It is a transmembrane protein that contains a leukine-rich repeat motif, a transmembrane domain and a cytoplasmic Toll/IL-1 receptor domain. The leukine-rich repeat motif is responsible for recognizing and binding with the pathogen-associated molecular patterns such as lipopolysaccharides (LPS) on the pathogens, hence initiating the innate immune response by recruiting downstream adapters such as myeloid-differentiation factor 88 (MYD88), TIR domain-containing adapter protein (TIRAP), TIR domain-containing adapter-inducing IFN-b (TRIF), TRIF-related adapter molecule (TRAM), and sterile-α and HEAT-Armadillo motif-containing protein.

Among all the human TLR homologs, TLR4 can be activated by long-chain saturated fatty acid. A study reveals a direct binding between palmitic acid and MD2, in which palmitic acid is associated with the hydrophobic pocket of MD2 [5, 6]. Subsequently, the binding between palmitic acid and TLR4/MD2 stimulates NFκB canonical pathway [6]. However, stearic acid or the monounsaturated oleic acid does not interact with TLR-4/MD-2 heterodimer [7]. The activation of TLR4 by long-chain saturated fatty acid seems to be exclusive for palmitic acid. The palmitic acid-activated TLR4 not only regulates the pro-inflammatory signaling pathways [8, 9], but also underlies the development of insulin resistance and leptin resistance in metabolic disorders [10]. A study shows that palmitic acid, by activating TLR4/MyD88 signaling pathway, recruits pro-inflammatory monocytes and macrophages to the islets and hence induces β-cell dysfunction in obese mouse models [10]. Another study shows that in the hypothalamus of the diet-induced obesity mouse model, activation of TLR4 by palmitic acid elicits inflammatory responses that result in the functional resistance to leptin [11]. However, whether palmitic acid affects TLR4 expression is not known.

TLR4 is overexpressed in colorectal cancer (CRC) [12, 13], and is directly correlated with the survival of the patients [12]. Experimental studies show that increased TLR4 activity promotes the growth, metastasis, and immune surveillance of CRC [14, 15]. In clinical biopsies, elevated TLR4 is associated with acute secretion of inflammatory cytokines such as interleukin 6 (IL6) and IL8 [13]. TLR4 also activates β-catenin pathway that promotes intestinal neoplasia in a PI3K-dependent manner [16]. Activation of TLR4 increases Akt phosphorylation [17] and hence increases the adhesiveness and metastatic capacity of the CRC by promoting β-1 integrin function [18] and upregulating Nox-1 expression [19]. Nevertheless, the oncogenic role of TLR4 is challenged by a study showing that high-fat diet (HFD) promotes the polarization of adipose tissue macrophages from M2 to M1 phenotypes, and TLR4 promotes adipose tissue macrophage phagocytosis and secretion of Cxcl10 that recruits T cells and inhibits the peritoneal seeding of CRC cells [20].

Here, we aimed to identify the critical regulator for CRC growth under HFD and explore the underlying mechanism of action. The finding is prerequisite for the development of effective therapeutic for CRC treatment.

## Results

### HFD feeding increases TLR4 expression in CRC

**Fig. 1:**
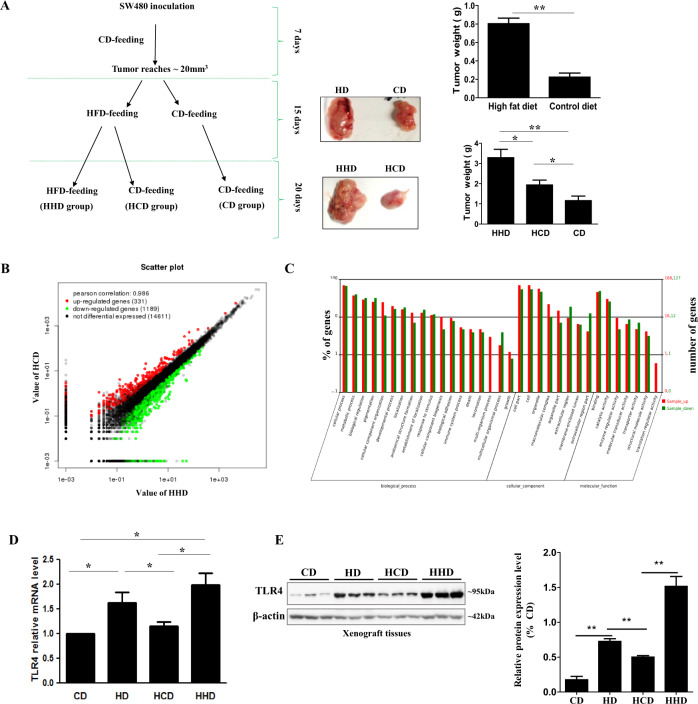
High-fat diet (HFD) feeding increases TLR4 expression in CRC. **A** Schematic diagram showing the dietary intervention and the tumor size and tumor weights of the xenograft mouse models. **B** RNA sequencing showing the number of upregulated and downregulated genes in the HCD and HHD tumor samples. **C** GO analysis for the biological functions of the genes that show significant changes between HCD and HHD tumor samples. **D** TLR4 mRNA levels and **E** the protein levels and quantification in the tumor tissues. CD, matched control diet feeding; HD, high-fat diet feeding; HHD, HFD feeding persisted for 35 days; HCD, HFD feeding for 15 days followed by CD feeding for 20 days. Data are shown as means ± SEM, *n* = 4–8 mice in each group. *p < 0.05, **p < 0.01 as indicated.

We first set up xenograft models by inoculating human CRC cell SW480 into the nude mice. After the tumors of the xenograft models reached about 20mm^3^ in size, we randomly divided the xenograft models into two groups, the HFD-feeding group (HD group) and the matched control diet-feeding group (CD group) (Fig. 1A). After 15-day dietary intervention, the tumor sizes and tumor weight of the HD group were significantly larger than CD group (Fig. 1A). To better identify the molecular mechanisms that regulate the CRC growth under HFD feeding, we randomly selected half of the mice in the HD group to have matched control diet feeding (HCD group), while the remaining mice kept feeding HFD (HHD group) for 20 days (Fig. 1A). Interestingly, we found that the tumor size and tumor weight in the HCD group were significantly less than those in the HHD group (Fig. 1A). The parallel concomitant changes in dietary intervention and the tumor sizes and tumor weight suggest that HFD feeding affects some reversible signaling mechanisms that influence the tumor growth.

We next examined whether changes in the genetic profiles of these tumor tissues may explain the enhanced CRC growth under HFD feeding. We performed RNA sequencing with the HHD and HCD tumor tissues. A total of 19707 genes were detected in the samples. Among these, 331 genes were upregulated, and 1189 genes were downregulated in the HHD samples when compared with HCD samples (Fig. 1B), implying that the dietary intervention affects the genetic profiles of the CRC. The Gene Ontology (GO) enrichment analysis revealed that most of the genes that were affected by the dietary intervention were involved in cellular process and metabolic process (Fig. 1C). Interestingly, among the 10 TLR homologs, HFD feeding increased TLR4 by 4-fold as indicated by the total read counts, which was in consistent with the FPKM (fragments per kilobase million)-normalized read counts that showed TLR4 was increased by 3.28-fold (Supplementary Table 1). Although the data also showed that HFD increased TLR3 by 20-fold as indicated by the total count, it had a discrepancy with the FPKM read count that showed HFD increased TLR3 by 38-fold. Furthermore, the null expression of TLR3 in CRC was not in agreement with other published studies [21, 22]. Therefore, we shortlisted TLR4 for the study and further validated its expressions in the tumor tissues by qPCR. Figure 1D shows that TLR4 mRNA levels were increased in the HD group, which was reduced in the HCD group but kept increasing in the HHD group. TLR4 protein levels also exhibited parallel concomitant changes with the dietary interventions (Fig. 1E). These in vivo data suggest that HFD increases TLR4 expressions in CRC.

### Palmitic acid increases TLR4 expression in CRC cells

**Fig. 2:**
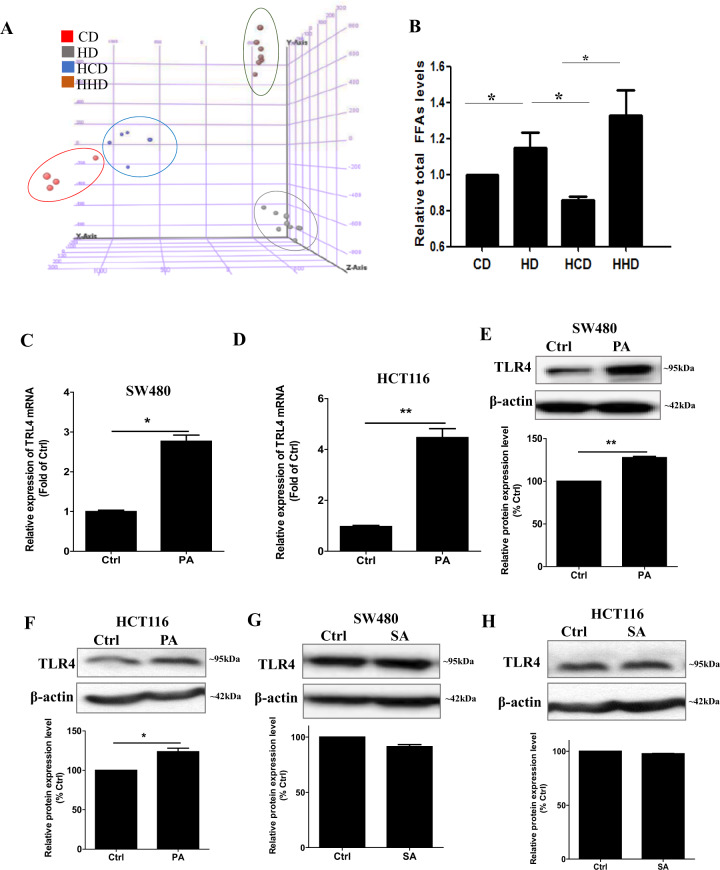
Palmitic acid increases TLR4 expression in CRC cells. **A** Principal component analysis (PCA) of the tumor samples in global lipidomics study. **B** Relative total free fatty acid (FFA) levels in the tumor tissues examined by the global lipidomics. Data are shown as means ± SEM, *n* = 4–8 mice in each group, *p < 0.05 as indicated. Relative expression levels of TLR4 mRNA in **C** SW480 and **D** HCT116 cells after palmitic acid (PA, 50 µM) treatments. Expression of TLR4 proteins in **E** SW480 and **F** HCT116 cells after palmitic acid (PA, 50 µM) treatments. Expression of TLR4 proteins in **G** SW480 and **H** HCT116 cells after stearic acid (SA, 50 µM) treatments. Fatty acid treatments were done in the presence of 1% fatty acid-free BSA, 1% fatty acid-free BSA alone served as control (Ctrl). Data are shown as means ± SEM, *n* = 3 independent experiments, *p < 0.05, **p < 0.01 as indicated.

The next question was, how HFD could affect TLR4 expressions in CRC. Since mice received high-fat dietary intervention, we studied the lipid profiles of the tumor tissues and examined whether the lipid changes would affect the TLR4 expressions. Our lipidomics data showed that the dietary intervention affected the tumor lipid profiles as revealed by the sample clustering in principal component analysis (Fig. 2A). HD group had higher total fatty acid levels when compared with CD group, the levels were reduced in the HCD group but kept increasing in the HHD group (Fig. 2B). The fatty acid species that showed the most significant changes were identified (Supplementary Table 2). Target lipidomics showed that, among all these identified fatty acid candidates, palmitic acid and stearic acid levels were elevated in the xenograft tissues of the HD and HHD group when compared with CD and HCD groups, respectively (Supplementary Table 3). Besides, these fatty acid levels were lower in the HCD group when compared with HD group (Supplementary Table 3). These data suggest that the palmitic acid and stearic acid levels in the tumor tissues are in parallel concomitant changes with the dietary interventions. These changes led us to investigate whether palmitic acid and stearic acid affected TLR4 expressions in CRC. We separately treated the CRC cells with palmitic acid or stearic acid. The data showed that palmitic acid significantly increased TLR4 mRNA levels in SW480 cells (Fig. 2C) and HCT116 cells (Fig. 2D). The treatments also significantly increased the TLR4 protein levels in these cells (Fig. 2E, F). However, stearic acid did not affect the TLR4 expression (Fig. 2G, H). These data suggest that HFD increases palmitic acid and stearic acid levels in CRC and only palmitic acid increases TLR4 expression.

### PU.1 expression that is critical for the palmitic acid-enhanced TLR4 expression

**Fig. 3:**
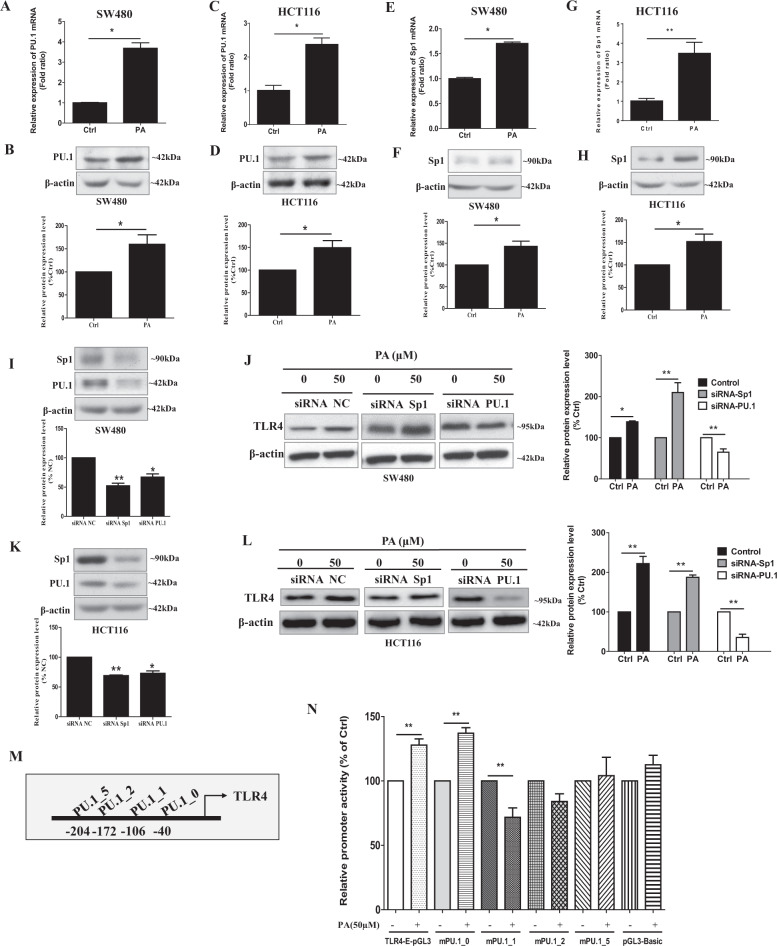
Palmitic acid increases PU.1 expression, mutations of PU.1-binding site on TLR4 promoter abolish the palmitic acid-increased TLR4 expression. After palmitic acid (PA, 50 µM) treatments, the relative **A** mRNA and **B** protein expressions of PU.1 in SW480 cells; the relative **C** mRNA and **D** protein expressions of PU.1 in HCT116 cells; the relative **E** mRNA and **F** protein expressions of Sp.1 in SW480 cells; the relative **G** mRNA and **H** protein expressions of Sp.1 in HCT116 cells. The protein expression of Sp.1 and PU1 in the **I** SW480 and **K** HCT116 cells after siRNA-mediated knockdown of Sp.1 and PU1, respectively. **J** TLR4 protein expressions and quantification in Sp1-knockdown or PU.1-knockdown SW480 cells after palmitic acid (PA, 50 µM) treatments. **L** TLR4 protein expressions and quantification in Sp1-knockdown or PU.1-knockdown HCT116 cells after palmitic acid (PA, 50 µM) treatments. **M** Schematic illustration for the PU.1 binding site on TLR4 promoter. **N** Effects of the PA treatment (PA, 50 µM) on TLR4 promoter activity, wild-type TLR4 promoter (TLR4-E-pGL3), and TLR4 promoter with mutations at PU.1-binding sites, mPU.1_0, mPU.1_1, mPU.1_2, and mPU.1_5. pGL3-Basic is control plasmid. Data are shown as means ± SEM. *n* = 3 independent experiments. *p < 0.05, **p < 0.01 as indicated. Fatty acid treatments were done in the presence of 1% fatty acid-free BSA, 1% fatty acid-free BSA alone served as control (Ctrl).

Since palmitic acid increased TLR4 mRNA levels, we investigated whether the acid affected TLR4 gene transcription. Other studies reported that TLR4 promoter has both Sp1 [23] and PU.1 [24, 25] transcription-regulatory elements. Interestingly, our data showed that palmitic acid treatment significantly increased the mRNA expressions of both PU.1 (Fig. 3A, C) and Sp1 (Fig. 3E, G) in the CRC cells. Palmitic acid also significantly increased the protein expressions of PU.1 and SP1 in these cells (Fig. 3B, D, F, H). To investigate whether Sp1 and PU.1 were involved in the palmitic acid-increased TLR4 expressions, we separately knockdown Sp1 and PU.1 in the CRC cells before palmitic acid treatments. Figure 3I, K shows the protein expressions of SP1 and PU.1 in the CRC cells after the siRNA-mediated knockdown. The results showed that the acid treatments significantly increased TLR4 expressions in Sp1-knockdown cells but not in the PU.1-knockdown CRC cells (Fig. 3J, L), suggesting that PU.1 is essential for the palmitic acid-increased TLR4 expression in CRC.

Lichtinger et al. have identified several PU.1-binding sites on the TLR4 promoter [25]. We next examined which sites were essential for the palmitic acid-mediated TLR4 transcription. We performed luciferase assay with TLR4 promoter constructs that have mutated PU.1-binding sites (mPU.1_0, mPU.1_1, mPU.1_2, and mPU.1_5) [25]. Figure 3M shows the PU.1-binding sites on the TLR4 promoter. Luciferase assay data in Fig. 3N showed that palmitic acid significantly increased the activity of the TLR4 promoter (−1 to −385 TLR4-E). However, mutations at PU.1_1, PU.1_2, and PU.1_5, but not at PU.1_0, abolished the palmitic acid-increased promoter activity (Fig. 3N). These data suggest that PU.1-binding sites at −106, −172, and −204 are essential for the palmitic acid-mediated TLR4 transcription.

### Palmitic acid promotes CRC cell proliferation in a TLR4-dependent manner

**Fig. 4:**
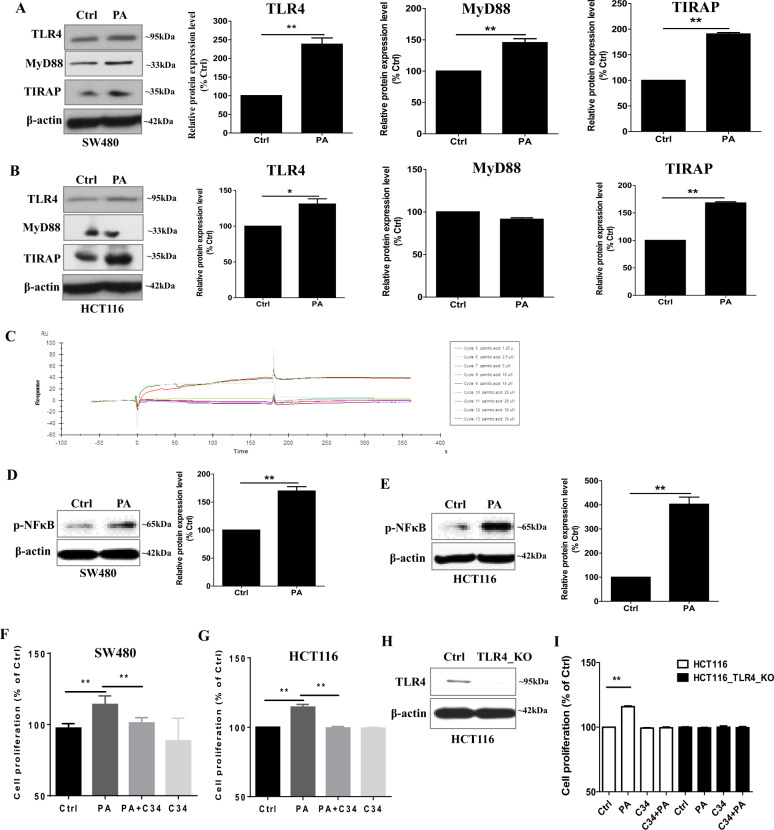
Palmitic acid increases CRC cell proliferation in a TLR4-dependent manner. Protein expression of TLR4, MyD88, and TIRAP in **A** SW480 and **B** HCT116 cells after palmitic acid (PA, 50 µM) treatments. **C** Kinetics analysis of palmitic acid binding to TLR4 protein based on SPR platform Biacore X100. Representative sensorgrams were obtained from injections of palmitic acid at different concentrations as indicated. Expression of p-NFκB in **D** SW480 and **E** HCT116 cells after palmitic acid (PA, 50 µM) treatments. Proliferation of **F** SW480 and **G** HCT116 cells after palmitic acid (PA, 50 µM) treatments in the presence or absence of C34. **H** Knockout of TLR4 in HCT116 cells (HCT116^TLR4-KO^). **I** Proliferation of HCT116 cells and HCT116^TLR4-KO^ cells after palmitic acid (PA, 50 µM) treatments in the presence or absence of C34. Data are shown as means ± SEM. *n* = 3 independent experiments. *p < 0.05, **p < 0.01 as indicated. Fatty acid treatments were done in the presence of 1% fatty acid-free BSA, 1% fatty acid-free BSA alone served as control (Ctrl).

Palmitic acid not only increased TLR4 expression but also the expressions of MyD88 and TIRAP in the CRC cells (Fig. 4A, B). However, palmitic acid did not bind to TLR4 as shown in the surface plasmon resonance (SPR) (Fig. 4C). In the SPR study, palmitic acid was injected sequentially with eight concentration gradients, which were 1.25, 2.5, 5, 10, 15, 20, 25, and 30 μM, and the 30 μM group was repeated after all the gradient injections to check if the protein-regeneration ability was good. As shown in Fig. 4C, the signals for the two 30 μM injections were almost overlapped, indicating that the protein-regeneration ability was good. The KD of the dissociation-equilibrium constant of palmitic acid binding to TLR4 protein was 35.56 M, which was obtained by computer fitting. The lower the KD value, the better the binding of the sample to the protein. The KD value suggests that there is less likely to have a binding between palmitic acid and TLR4 protein, which agrees with other studies [5, 6, 26]. The enhanced phosphorylation of NFκB in CRC cells after palmitic acid treatment (Fig. 4D, E) suggests that the activation of the TLR4 signaling pathway is, at least in part, due to the enhanced TLR4, MyD88, and TIRAP expressions in the cancer cells but not a direct binding of palmitic acid to the TLR4 protein.

Our data also showed that palmitic acid increased CRC cell proliferation that was abolished in the presence of TLR4 inhibitor C34 (Fig. 4F, G). To further suggest that TLR4 mediated the palmitic acid-increased CRC cell proliferation, we knock out TLR4 in the HCT116 cells (TLR4_KO) (Fig. 4H). Data showed that palmitic acid failed to increase the proliferation of the TLR4_KO cells (Fig. 4I). These data evidently suggest that palmitic acid increases CRC cell proliferation in a TLR4-dependent manner.

### HFD or palmitic acid-rich diet (PAD) increases CRC growth in a TLR4-dependent manner

**Fig. 5:**
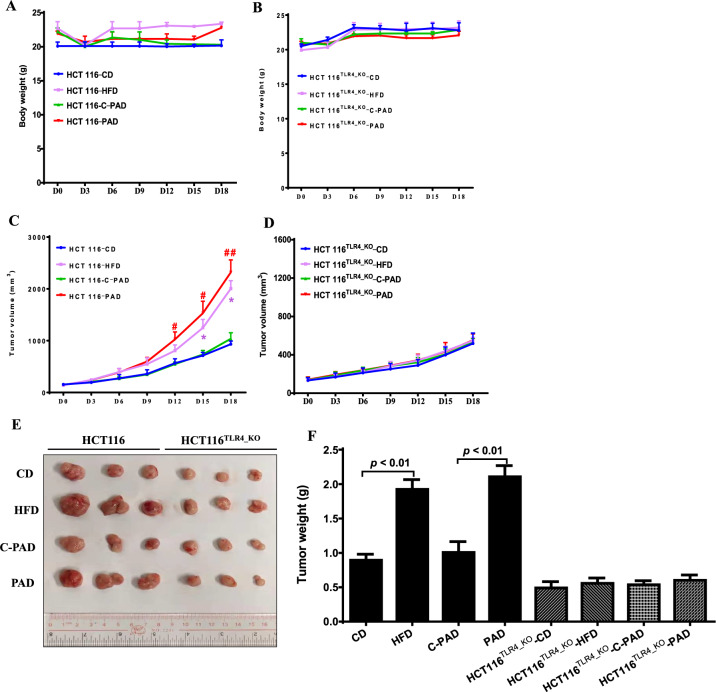
HFD or palmitic acid-rich diet (PAD) increases CRC growth in a TLR4-dependent manner in vivo. The body weight of the xenograft mouse models that were inoculated with A wild-type HCT116 cells or **B** HCT116^TLR4-KO^ cells, during the 18-day dietary intervention. The tumor volume of the xenograft mouse models that were inoculated with **C** wild-type HCT116 cells or **D** HCT116^TLR4-KO^ cells, during the 18-day dietary intervention. **E** Pictures of the dissected tumor tissues. **F** The tumor weight of the xenograft mouse models that were inoculated with wild-type HCT116 cells or HCT116^TLR4-KO^ cells, after the dietary intervention. HFD high-fat diet, CD matched control diet for HFD, PAD palmitic acid-rich diet, C-PAD matched control diet for PAD. Data are shown as means ± SEM, *n* = 3 mice in each group. *p < 0.05 compared with CD, ^#^*p* < 0.05, ^##^*p* < 0.01 compared with C-PAD.

Next, we examined whether TLR4 mediated the HFD-associated CRC growth in vivo. We subcutaneously inoculated wild-type HCT116 cells or TLR4-knockout HCT116 cells (HCT116^TLR4_KO^) into the nude mice. When the tumor volume reached ~80 mm^3^ in volume, we randomly divided them into HFD group and CD group. The HFD (D12492, Research Diets) was formulated to have 49.9 g of palmitic acid in 254.5 g of the total fatty acids, in which palmitic acid accounts for 19.6% of the total fatty acids. The mice in the control group were fed a matched control diet (CD) (D12450J, Research Diets). Although HFD is commonly used to induce obesity, it cannot clearly demonstrate the effect of palmitic acid on CRC growth in the mouse model. Therefore, another batch of mice were fed a palmitic acid-rich diet (PAD) (D16042106, Research Diets), which contains 106.5 g of palmitic acid in 262.8 g of fatty acids, in which palmitic acid accounts for 40.5% of the total fatty acids. The control mice were fed the corresponding matched control diet (C-PAD) (D17042705, Research Diets). No apparent difference in the food intakes was observed among these groups. The diets did not significantly affect the body weight of the mice during the 18-day dietary intervention (Fig. 5A, B). However, for the mice that were inoculated with the wild-type HCT116 cells, both HFD and PAD significantly increased the tumor volume (Fig. 5C, E) and tumor weight (Fig. 5F) when compared with CD and C-PAD, respectively. Interestingly, for the mice that were inoculated with the HCT116^TLR4_KO^ cells, both HFD and PAD failed to increase the tumor volume (Fig. 5D, E) and tumor weight (Fig. 5F). These data suggest that HFD and PAD increase CRC growth in a TLR4-dependnet manner in vivo.

### TLR4 does not affect the tumor-infiltrating immune cell profiles under HFD conditions

Another study reported that TLR4 activated the inflammatory pathways and promoted the development of colitis-associated colorectal tumors [15]. Here, we used CT26 cells and BALB/c mice to establish the CRC-bearing mouse model to examine whether TLR4 affects the tumor-infiltrating immune cell profiles under HFD conditions. As shown in Supplementary Fig. S1A, HFD did not significantly affect the tumor-infiltrating immune cell profiles, such as natural killer (NK) cells, cytotoxic T cells, and M1 and M2 macrophages. To understand how TLR4 promoted CRC growth under HFD, we examined the tumor-infiltrating immune cell profiles in the mice that had tumor TLR4 activity inhibited by C34. As shown in Supplementary Fig. S1B, C, inhibition of TLR4 activity in the tumor did not affect the relative percentages of M1 and M2 macrophages under different dietary interventions, it reduced the relative NK and cytotoxic T-cell populations in the tumor under HFD feeding, but not PAD feeding, when compared with the respective matched control diet. In CRC, cytotoxic T cells and NK cells are known to have antitumor abilities [27, 28], the reduced NK and cytotoxic T-cell populations should promote tumor growth. These data suggest that tumor-infiltrating immune cells, such as cytotoxic T cells, NK cells, and macrophages, are not involved in the regulatory role of TLR4 in promoting CRC growth under HFD-feeding conditions.

### TLR4 regulates CRC metabolism under HFD conditions

**Fig. 6:**
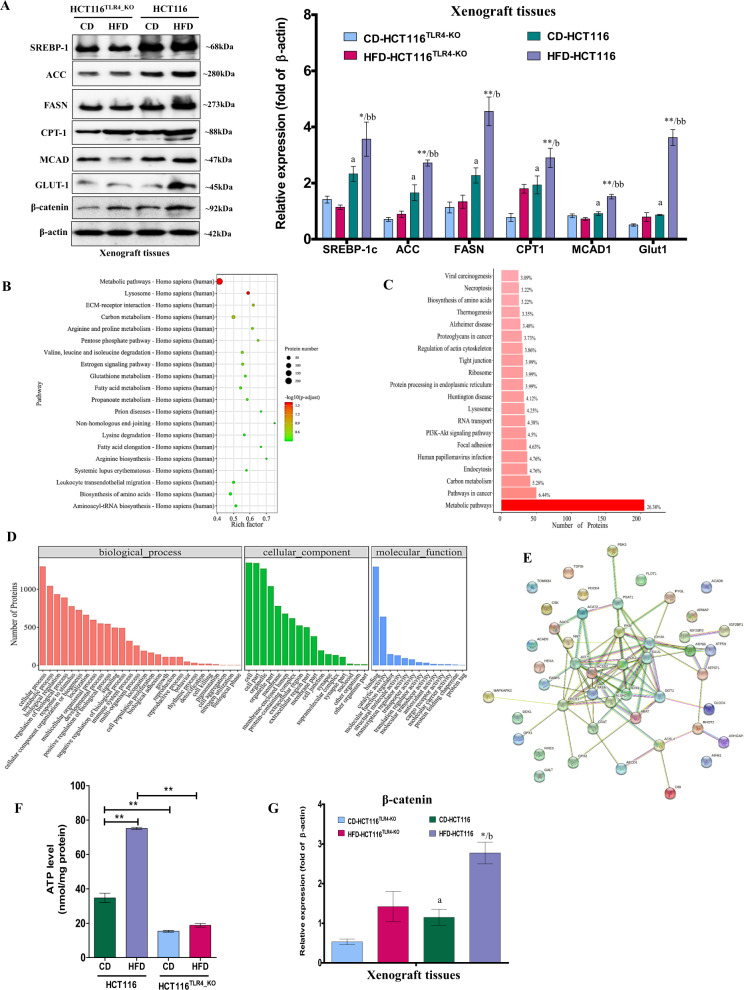
Knockout of TLR4 in CRC reprograms cancer metabolism in vivo. **A** Protein expression of SREBP-1c, ACC, FASN, CPT-1, MCAD, GLUT-1, and β-catenin in the tumor tissues of mouse models inoculated with wild-type HCT116 cells or HCT116^TLR4-KO^ cells under different dietary interventions. **B–D** In iTRAQ proteomics study, the gene ontology (GO) pathway enrichment analysis and Kyoto Encyclopedia of Genes and Genomes (KEGG) pathway analysis of the biological functions of the differentially expressed proteins in the wild-type HCT116 or HCT116^TLR4-KO^ tumor tissues dissected from HFD mice. **E** STRING analysis of the highlighted metabolic enzymes that show significant differences between the wild-type HCT116 or HCT116^TLR4-KO^ tumor tissues. **F** ATP levels of the tumor tissues of the mouse models inoculated with wild-type HCT116 cells or HCT116^TLR4-KO^ cells under the dietary intervention. **G** Quantification of the β-catenin expressions in the tumor tissues of mouse models inoculated with wild-type HCT116 cells or HCT116^TLR4-KO^ cells under different dietary interventions. Data are shown as means ± SEM. *n* = 4 and 5 mice in each group. *p < 0.05, **p < 0.01 compared with CD-HCT116, *a* < 0.05 compared with CD-HCT116^TLR4-KO^, *b* < 0.05, *bb*<0.01 compared with HFD-HCT116^TLR4-KO^. CD-HCT116^TLR4-KO^ mice inoculated with TLR4-KO HCT116 cells had CD diet, HFD-HCT116^TLR4-KO^ mice inoculated with TLR4-KO HCT116 cells had HFD diet, CD-HCT116 mice inoculated with HCT116 cells had CD diet, HFD-HCT116 mice inoculated with HCT116 cells had HFD diet. ACC acetyl-CoA carboxylase, FASN fatty acid synthase, CPT1 carnitine palmitoyltransferase-1, MCAD medium-chain acyl-CoA dehydrogenase, GLUT1 glucose transporter, SREBP-1c sterol regulatory element-binding transcription factor-1.

Next, we explored whether TLR4 promoted CRC growth under HFD conditions by regulating cancer metabolism. As shown in Fig. 6A, knockout of TLR4 in the tumor tissues abolished the HFD-enhanced expressions of acetyl-CoA carboxylase (ACC), fatty acid synthase (FASN), carnitine palmitoyltransferase-1 (CPT-1), medium-chain acyl-CoA dehydrogenase (MCAD), glucose transporter GLUT1, and also sterol regulatory element-binding transcription factor-1 (SREBP-1c), the master transcription factor that regulates cellular lipogenesis and lipid homeostasis [29]. iTRAQ (isobaric tags for relative and absolute quantitation)-based proteomics with the tumor tissues also showed that knockout of TLR4 changed the metabolism of CRC (Fig. 6B–D) and also regulated other metabolic enzymes (column KO-HFD/HFD in Supplementary Table 4A) that the changes of many of these enzymes were not due to the dietary intervention (KO-HFD/KO-CD column in Supplementary Table 4A). Further analysis showed that these metabolic enzymes were either located in mitochondria or cytoplasm (Supplementary Fig. S2A), with catalytic activities (Supplementary Fig. S2B). More importantly, they were mainly involved in energy production (Supplementary Fig. S2C) and took part in triglyceride biosynthesis, glucose metabolism, fatty acid activation, fatty acid, triglyceride, and ketone synthesis (Supplementary Fig. S2D) in a cooperative manner, as illustrated in the STRING analysis (Fig. 6E), suggesting that the regulatory role of TLR4 on CRC metabolism under HFD conditions is not attributed to a single metabolic enzyme but a collaborative work of the enzymes. Indeed, changes of the metabolism affected ATP production as shown in Fig. 6F that knockout of TLR4 abolished the HFD-increased ATP level in the tumor tissues. Interestingly, our data also showed that knockout of TLR4 abolished the HFD-enhanced β-catenin expressions in the tumor tissues (Supplementary Table 4B and Fig. 6G), suggesting that the energy homeostasis regulated by β-catenin [30] in CRC under HFD conditions is in relation to TLR4 activity. Taken together, these data suggest that TLR4 regulates the CRC growth under HFD by programming the cancer metabolism.

## Discussion

Our data clearly demonstrate that HFD feeding increases palmitic acid levels in CRC, which in turn increases TLR4 expressions by increasing PU.1 level and PU.1-dependent TLR4 gene transcription. The increased TLR4 expression and activity mediate the CRC growth under HFD. Knockout of TLR4 in CRC completely abolishes the HFD-enhanced CRC growth both in vitro and in vivo. TLR4 expression does not affect the tumor-infiltrating immune cell profiles but regulates the expressions of the metabolic enzymes and ATP production that underlie how TLR4 promotes the CRC growth under HFD conditions.

It is generally believed that obesity promotes CRC progression. Negative associations between visceral adipose tissues and survival are frequently observed in the CRC patients [31]. Some studies report an absence of a direct linkage between obesity and CRC, but an indirect linkage between the chronic inflammation secondary to obesity and CRC [32]. Indeed, inflammation is one of the factors that promotes CRC growth under obesity conditions [33]. It has been reported that TLR4 signaling pathway activates TNF-α and NFκB that induce inflammation and promote CRC growth [15]. TLR4 also elevates the production of prostaglandin E2 and cyclooxygenase-2 in the inflammation-associated colorectal neoplasia in humans and mice [15]. MyD88 is important in facilitating the growth of intestinal polyps in Apc^min/+^ mice [34]; the roles of TLR/MyD88 signaling pathway in CRC pathogenesis mainly focus on inflammation. However, under HFD, dietary fat suppresses the peritoneal seeding of CRC cells, which is mediated by TLR4/Cxcll10 axis in the adipose tissue macrophages [20]. We have also investigated whether TLR4 would affect the tumor-infiltrating immune cells. However, our results suggest that knockout of TLR4 does not have significant effect on the percentages of cytotoxic T cells, and M1 and M2 macrophages in the tumor tissues. Whereas knockout of TLR4 reduces the significant reduction of the NK cell percentage under HFD conditions. A study suggests that activation of TLR4 inhibits NK cell function [35]. It deserves further investigation to examine whether under HFD conditions, activation of TLR4 reduces NK cell infiltration into the tumor tissues, which may also suggest how HFD promotes CRC growth.

Nevertheless, inflammation alone is not sufficient to increase CRC growth, but contributions from other factors are equally important in the pathogenesis. A recent study shows that TLR4 induces inflammation by reprogramming macrophage metabolism, in which the abundances of phosphatidylcholines and phosphatidylethanolamines in the bone marrow-derived macrophages are significantly changed [26]. Our data show that TLR4 expression is directly associated with the metabolism of the CRC. Knockout of TLR4 in the xenograft tissues changes the expressions of the metabolic enzymes. These enzymes are mainly involved in energy production, triglyceride biosynthesis, glucose metabolism, fatty acid activation, fatty acid, triglyceride, and ketone synthesis, IGF, and insulin pathways. These metabolic enzymes are working in a cooperative manner as illustrated in the STRING analysis. Therefore, the effects of TLR4 on the CRC metabolism are not attributed to a single metabolic enzyme or a metabolic pathway. Our study suggests that TLR4 reprograms CRC metabolism that may affect the cancer growth, this notion agrees with our findings that HFD fails to increase the growth of the TLR4-knockout CRC. The regulatory role of TLR4 in CRC metabolism has also been reported in which TLR4 siRNA inhibits proliferation and invasion of CRC by downregulating acyl coenzyme A cholesterol acyltransferase 1 (ACAT1) [36]. ACAT1 is an intracellular enzyme that catalyzes the formation of cholesterol ester from free cholesterol and long-chain fatty acids. Besides, TLR4 also increases GSK3β phosphorylation and the related lipogenic enzymes hence increase the metastatic ability of CRC [37].

Studies show that palmitic acid is a ligand for TLR4 [5, 6]. However, a recent study using molecular dynamics simulations demonstrates that the presence of palmitate molecules within the active TRL4/MD2 receptor complex will destabilize the complex [26]. Furthermore, palmitic acid does not induce TLR4 dimerization or endocytosis [26]. Our data on the surface plasmon resonance (SPR) suggest that palmitic acid is less likely binding onto TLR4 protein. The activation of TLR4/NFκB signaling pathway upon palmitic acid challenge may be due to the elevated expressions of the TLR4 in the cancer cells. The physiological or pathogenic factors that increase TLR4 expression are less reported. A study shows that LPS does not affect TLR4 expression [38, 39], but increases the phosphorylation of ERK1/2 and p38 and activates NFκB [38]. Another study demonstrates that continuous LPS stimulation reduces TLR4 expression [40]. Other factors like endotoxins do not affect TLR4 promoter activity but decrease the TLR4 steady-state mRNA levels by increasing the turnover rate of the TLR4 transcripts [41]. Hypoxic condition in the inflamed intestinal lining induces TLR4 expressions [42]. Interestingly, our study suggests that palmitic acid enhances PU.1 transcription factor expression, and mutations of the PU.1 binding site at −106, −172, and −204 on TLR4 promoter completely abolish the palmitic acid-mediated TLR4 transcription. PU.1 is a critical transcription factor in TLR4 transcription, which increases the recruitment of polymerase II to the proximal TLR4 promoter and directs the location of the transcription-initiation complex [25]. It deserves further investigation to study how the palmitic acid increases PU.1 expression in CRC, and whether palmitic acid facilitates the physical interaction between PU.1 and the TATA-box-binding protein TFIID [43] or other critical trans-acting elements that enhance the TLR4 transcription.

Inhibiting TLR4 signaling seems to be a pragmatic therapeutic approach for CRC or obesity-associated CRC treatment. Indeed, inhibition of the TLR4 signaling pathway by TIPE2 inhibits the TLR4-mediated CRC development by regulating the TLR4 inflammatory effect and inhibiting further amplification of the cascade reactions [44]. miRNA 6869-5p-targeting TLR4/NFkB signaling acts as a tumor suppressor in CRC [45]. Our study also shows that knockout of TLR4 and inhibiting the TLR4 signaling pathway completely abolish the HFD-associated CRC growth. Therefore, TLR4 antagonists and also the negative regulator of the adapter complex may be the novel therapeutics for cancer treatments. Examples of these endogenous molecules are the flightless I-like homolog (Fliih) that negatively disrupts the TLR4–MyD88 complex [46], sterile-α and armadillo–motif–containing protein (SARM) that inhibits the TRIF complex formation by directly binding to TRIF [47]. Besides, clinically used therapeutic agents that are used to control excessive inflammation by inhibiting TLR4 signaling may be developed as obesity-associated CRC therapeutics, these include TAK-242, Candesartan, Valsartan, Fluvastatin, Simvastatin and Atorvastatin [48].

On the contrary, TLR4 not only involves in innate immune system but also activates adaptive immune system in response to cancer. TLR agonizts have been approved by the Food and Drug Administration for cancer and cancer-related conditions such as Bacillus Calmette–Guérin (BCG) that targets both TLR4 and TLR2 for the treatment of superficial transitional bladder cell carcinoma; monophosphoryl lipid A as adjuvant to Cervarix^®^ for the prophylaxis of HPV-associated cervical cancer, and Imiquimod targets TLR7 for treating basal cell carcinoma and actinic keratosis [49]. However, TLR4 ligand for CRC treatment has not been developed. A study shows that among the TLR4 ligands LPS and Taxol, only Taxol inhibits the growth of CRC cells in vitro [50]. Nevertheless, in the in vivo model, Taxol does not have any significant inhibitory effect on CRC growth, and a combination of Taxol and LPS even accelerates the cancer growth [48]. These findings suggest that using TLR4 agonist to mediate both CRC inhibition and activation of immune effector requires more in-depth studies and validation.

In conclusion, our data demonstrate that the TLR4 expression in CRC is increased by palmitic acid in PU.1-dependent manner. Knockout of TLR4 completely abolished the HFD-enhanced CRC growth by re-programming the cancer metabolism and reducing ATP production. Our study has identified a critical regulator of CRC growth under HFD conditions, and provides evidence to support the development of TLR4-targeting therapeutics for cancer treatment.

## Materials and methods

### Reagents and chemicals

Palmitic acid (PA), stearic acid (SA), and fatty acid-free bovine serum albumin (BSA) were purchased from Sigma–Aldrich. C34, MG132 and actinomycin D were purchased from Caymen Chemicals. Antibodies against SREBP1 (ab28481), MCAD (ab92461), TLR4 (ab13556), TIRAP (ab17218), MyD88 (ab135693), and β-actin (ab6276) were purchased from Abcam. Antibodies against Sp1 (#9389), PU.1 (#2266), ACC (#3662), FASN (#3180), CPT1A (#97361), GLUT1 (#73015), p-NFκB (#3033), and β-catenin (#8480) were purchased from Cell Signaling. Horseradish peroxidase (HRP)-conjugated secondary antibodies (sc2357) were purchased from Santa Cruz Biotechnology. TLR4 promoter reporters were given as a kind gift by Professor Michael Rehli at the University of Regensburg Medical School, Germany. All the diets, including high-fat diet (HFD, D12492) and its matched control diet (CD, D12450J), palmitic acid-rich diet (PAD, D16042106) and its matched control diet (C-PAD, D17042705), were purchased from Research Diets, Inc.

### Cell lines and culture

CRC cell lines HCT116, SW480, and CT26 were purchased from American Type Culture Collection (ATCC) and were authenticated. The cell lines were tested for mycoplasma contamination. Cells were cultured in Dulbecco’s modified essential medium supplemented with 10% fetal calf serum (Life Technologies Ltd), and 1% penicillin and streptomycin at 37 °C in a humidified incubator with 5% CO_2_.

### Cell proliferation

CRC cells were seeded in a 96-well plate and grew for 24 hours. Then, the cells were treated with 50 μM palmitic acid (PA) or stearic acid (SA) for 48 hours in DMEM medium containing 1% fatty acid-free BSA. In all, 1% fatty acid-free BSA alone served as control. For C34 treatment, cells were preincubated with 5 μM C34 in 1% fatty acid-free BSA-containing medium for 3 hours, and then treated with or without PA (50 μM). Cell proliferation was measured by Cytoquant kit (Invitrogen) as per the manufacturer’s instructions.

### siRNA transfection

siRNA transient transfection was performed using Lipofectaine RNAiMAX (Invitrogen) transfecting reagent according to the manufacturer’s instructions. Briefly, CRC cells were seeded in a six-well plate and transfected with 5pmol/well of the siRNA with 5 μL of Lipofectamine RNAiMAX for 24 hours. The siRNA-targeting sequence of PU.1 was “CCCUAUGACACGGAUCUAUTT”. The siRNA-targeting sequence of Sp1 was “CCUCACAGCCACACAACUUTT”. The sequence of the negative control siRNA was “UUCUCCGAACGUGUCACGUTT”.

### Establishment of TLR4-knockout cells

TLR4 CRISPR–Cas9 (Santa Cruz) and TLR4 HDR (Santa Cruz) plasmids were used for the TLR4-knockout experiments in HCT116 cells (HCT116^TLR4-KO^). HCT116 cells were seeded in six-well plates and transfected with the CRISPR plasmids using UltraCruz transfection reagent (Santa Cruz). The control cells were transfected cells with pX330-U6-Chimeric_BB-CBh-hSpCas9 (HCT116^TLR4-KO^-Ctrl). After 48 hours, the transfected cells were treated with 1 μg/mL puromycin (Sigma) for 3–4 weeks for the selection of TLR4-knockout stable clones. HCT116 cells with TLR4 gene knockout were confirmed by Western blot analysis.

### HCT116-bearing xenograft mouse model

Male nude mice (4–5 weeks old) were purchased from the Laboratory Animal Services Centre, Chinese University of Hong Kong, housed in ventilated cages in the animal room at the Hong Kong Baptist University, with a 12-h light–dark cycle and free access to food and water. To set up the xenograft mouse models, 1 × 10^6^ of the wild-type HCT116 cells, HCT116^TLR4-KO^ cells, or the control for HCT116^TLR4-KO^ cells were subcutaneously inoculated into the right flank of mice. When the tumors were grown to 80–100 mm^3^ in size, mice were randomly divided into 4 groups (*n* = 3 mice for each group) and fed with HFD or its matched control diet CD, or PAD or its matched control diet C-PAD for 18 days. The tumor volume and body weight were monitored every day. Tumor volume was measured by a caliper and calculated according to the formula: (length × width^2^)/2. No blinding was done. After 18 days of the dietary intervention, mice were sacrificed, and tumors were dissected, weighed, and stored at −80 °C for subsequent experiments.

### CT26-bearing xenograft mouse model

Male Balb/c mice (4–5 weeks old) were purchased from the Laboratory Animal Services Centre, Chinese University of Hong Kong, housed in ventilated cages in the animal room at the Hong Kong Baptist University, with a 12-h light–dark cycle and free access to food and water. To set up the CT26-bearing xenograft mouse model, 5 × 10^5^ of CT26 cells were subcutaneously inoculated into the left flank of mice. When the tumors were grown to 80–100 mm^3^ in size, mice were randomly divided into 6 groups (*n* = 4 mice for each group). Mice were fed with HFD or its matched control diet CD, or PAD or its matched control diet C-PAD, with or without daily intratumoral injection of C34 (1 mg/kg) [51, 52]. No blinding was done. After 18 days of the dietary intervention, mice were sacrificed, and tumors were dissected for subsequent experiments.

### Real-time quantitative polymerase chain reaction (qPCR)

Total RNA was extracted from the samples using Trizol reagent (Invitrogen) and treated with DNAse 1 (Invitrogen). RNA (1 μg) was reverse-transcribed into cDNA using PrimeScript Reverse Transcriptase reagents (Takara Bio). qPCR was performed using SYBR green reagents in ABI 7500 fast real-time PCR system (Applied Biosystems). All reactions were carried out in triplicates under the following cycling conditions: 50 °C for 2 min, 95 °C for 10 min, 40 cycles of 95 °C for 15 s, and 60 °C for 1 min. The relative mRNA expressions were normalized using β-actin as an internal control. Negative 2ΔΔCt method was applied to calculate the fold changes of the gene expressions. The primers used in this study were TLR4 forward: 5- CGAGGAAGAGAAGACACCAGT-3, TLR4 reverse: 5-CATCATCCTCACTGCTTCTGT-3; Sp1 forward: 5-GGCTACCCCTACCTCAAAGG-3, Sp1 reverse: 5-CACAACATA CTGCCCACCAG-3; PU.1 forward: 5-GTGCCCTATGACACGGATCT-3, PU.1 reverse: 5-GAAGCTCTCGAACTCGCTGT-3; β-actin forward: 5-GCACCACACCTTCTACAATG-3 and β-actin reverse: 5-TGCTTGCTGATCCACATCTG-3.

### Western blot analysis

Cells were collected and suspended in lysis buffer containing 150 mM sodium chloride, 1% Triton X-100, 0.5% sodium deoxycholate, 0.1% SDS, 50 mM Tris (pH 8.0), and protease and phosphatase inhibitors, incubated on ice for 30 min, and then centrifuged for 20 min (14,000 rpm) at 4 °C. Protein samples were denatured for 10 mins at 95 °C. The protein sample of 10–40 μg was separated on 10% sodium dodecyl sulfate–polyacrylamide (SDS-PAGE) gel and transferred onto polyvinylidene difluoride (PVDF) membranes. The transferred proteins were incubated overnight at 4 °C with the corresponding primary antibody at 1:1000 ratio. Immunodetection was accomplished using horseradish peroxidase-conjugated secondary antibody, followed by enhanced chemiluminescence (ECL) detection system (Amersham). β-actin served as a loading control for each sample.

### TLR4 promoter reporters and dual-luciferase reporter assay

Human TLR4 promoter reporters were the kind gifts from Prof Michael Rehli at the University of Regensburg Medical School. HCT116 cells were seeded in a 24-well plate and were separately transfected with the TLR4 promoter reporter (TLR4-E, mPU.1_0, mPU.1_1, mPU.1_2, mPU.1_5, and pGL3-Basic) using Lipofectamine^TM^ 3000 Transfection Reagent (Thermo Fisher Scientific) for 8 h, and then treated with or without PA (50 μM) in the presence of 1% fatty acid-free BSA for 48 h. Cells were lysed with 100 μL of lysis buffer and 20 μL of cell lysate was subjected to Dual Luciferase Assay (Beyotime Biotechnology). The firefly luciferase signals were normalized to that of Renilla luciferase by EnVision Mutilabel Reader. The luciferase reporter activity was calculated, all values were expressed as fold induction relative to basal activity.

### Surface plasmon resonance (SPR) biosensor analysis

The binding affinity of baicalein to TLR4 in vitro was assayed using the SPR spectroscopy (Biacore X100 System, GE Healthcare). TLR4 protein (molecular mass, 70.5 KDa) was purchased from Sino Biological. The TLR4 protein was immobilized on a CM5 sensor chip according to the standard procedures. The data were collected at a constant 1‰ DMSO–PBS flow rate of 30 μl/min at 25 °C. Palmitic acid was dissolved with 1‰ DMSO in PBS buffer, and added in mobile phase as analyte. The eight concentration-gradient stocks of palmitic acid were 1.25, 2.5, 5, 10, 15, 20, 25, and 30 µM. Samples were added at 60 s and dissociated at 180 s. The association (Kon) and dissociation (Koff) rate constants and the equilibrium-dissociation constant (KD, Koff) were calculated using Biacore X100 evaluation software with 1:1 Langmuir binding fitting model applied.

### Fluorescence-activated cell sorting (FACS) of the tumor-infiltrating immune cells

CT26-bearing mouse models were used for this experiment. Mice were anesthetized by isoflurane. Tumors were dissected and kept in serum-free medium. Liberase DL solution (Roche), Liberase TL solution (Roche), and DNase I were added to the samples, mixed, and incubated for 45 min at 37 °C under continuous shaking. Tumor cells were filtered using a 100-μm cell strainer, centrifuged, and suspended in 1% w/v BSA containing PBS. FACS was performed with the flow cytometer (BD FACSCalibur system). BD FACSVia Research Loader Software was used to analyze the data. NK cells were CD3^−^CD49b^+^NCR1^+^, cytotoxic T cells were CD3^+^CD8^+^. The tumor-associated macrophages were isolated from the tumors using Anti-F4/80 Microbeads Ultrapure Kit (Miltenyi Biotec). M1 macrophages were CD80^+^, M2 macrophages were CD206^+^.

### LC/MS-based lipidomics analysis

The xenograft tissues (200 mg) from each mouse were homogenized by sonication in 2 ml of PBS and 0.4 ml of solvent A (1 M NaCl, 0.1% HCl). The lipid samples were extracted twice by adding Folch reagent (chloroform/methanol: 2/1). After centrifugation at 2000 × *g* for 30 mins at 4 °C, the lipid sample (the lower phase) was collected and evaporated under nitrogen stream. The residue was reconstituted in 100 μL of isopropanol–acetonitrile (1:9, v/v) for liquid chromatography mass spectrometry (LC/MS) analysis. The LC/MS-based lipidomics analysis and data processing were performed by an Agilent 6540 UHD Accurate-Mass Q-TOF LC/MS mass spectrometer (Agilent Technologies), which was connected to an Agilent 1290 Infinity UHPLC *via* an ESI ion source for total lipid analysis. The Agilent 6450 Triple Quadrupole LC/MS system, accompanied with MassHunter Workstation software (Version B.04.00 Qualitative Analysis, Agilent Technologies), was connected to an Agilent 1290 Infinity UHPLC to quantify the concentrations of targeted fatty acids.

### RNA sequencing

Total RNA was extracted from xenograft tissues, and the RNA was quantified by Nanodrop. Purity of samples was checked on agarose gels by evaluating the 28 S and 18 S ribosomal RNA bands. The RNA samples were sent to GENE DENOVO Company for sequencing. RNA integrity number (RIN) was measured on Agilent Bio Analyzer 2100 system. Raw sequencing data were analyzed by FastQC program. Clean reads to the genome were aligned before performing normalization and statistical modeling to identify DEGs. R packages edgeR and DESeq2 were used to identify the gene differences. Enrichr web-based tool was used to perform gene set enrichment analysis to identify the biological functions of the up- or downregulated genes with focus on the gene ontology (GO) terms and Kyoto Encyclopedia of Genes and Genomes (KEGG) pathways using a standard false discovery rate (FDR) of <0.05.

### iTRAQ (isobaric tags for relative and absolute quantitation)-based proteomics analysis

Proteins from xenograft tissues were sent to Fitgene Biotech Company for iTRAQ proteomics study. The proteins were extracted before proteolysis and labeled with iTRAQ reagents. Peptides were separated and inspected by LC–MS/MS. The obtained raw data were analyzed by SEQUEST engine, Proteome Discoverer 1.4 (Version 1.4.0.288 Thermo Fisher) software and ProteinPilot™ software (4.5 version 1656, AB Sciex). Biological functions of the differentially expressed proteins were identified by the Database for Annotation, Visualization and Integrated Discovery, Gene Ontology (GO), and Kyoto Encyclopaedia of Genes and Genomes (KEGG) analyses. The highlighted proteins were put into STRING (version 11.0) for protein-interaction analysis. A network for protein–protein interaction and gene neighborhoods was provided by STRING, protein coregulation was provided by ProteomeHD.

### Statistical analysis

We used SPSS software to perform statistical analysis of the data, one-way analysis of variance (ANOVA) to examine the variance within each group and the significant difference between groups with *p < 0.05, **p < 0.01, and **p < 0.001. The data are shown as the mean ± SE, *n* = 3 independent experiments.

## Supplementary information

Supplementary Figures

Supplementary Tables

Reproducibility Checklist form

Detailed Attribution of Authorship

## Data Availability

It does not apply for the paper
