# Supplementary material for: Toll-like receptor 4 is a master regulator for colorectal cancer growth under high-fat diet by programming cancer metabolism
Source: Cell Death Dis. 2021 Aug 12;12(8):791. doi: 10.1038/s41419-021-04076-x (PMC8360949; doi:10.1038/s41419-021-04076-x)
Supplement: Supplementary file 1 — Supplementary Figures [file 41419_2021_4076_MOESM1_ESM.pdf]

Supplementary Figure S1

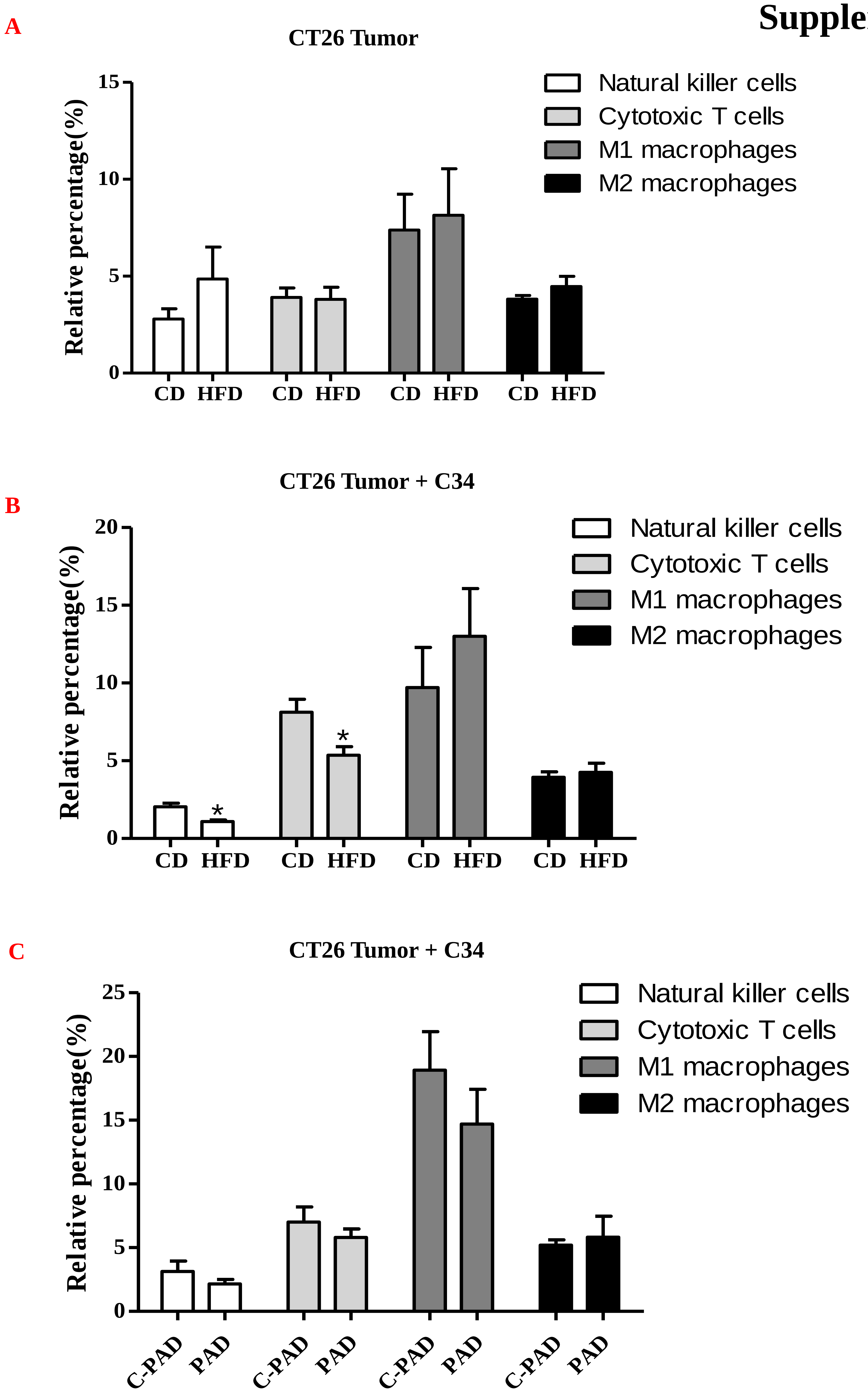

Supplementary Figure S1. Tumor-infiltrating immune cell profiles under different dietary interventions. Fluorescence-activated Cell Sorting (FACS) showing the percentage of natural killer (NK), cytotoxic T cells, M1 and M2 macrophages in the (A) CT26 tumor and (B) CT26 tumor with daily intratumoral injection of C34 (1mg/kg), under HFD or CD feeding for 18 days, and in the (C) CT26 tumor with daily intratumoral injection of C34 (1mg/kg) under PAD or C-PAD feeding for 18 days. NK cells are CD3-CD49b+NCR1+, cytotoxic T cells are CD3+CD8+, M1 macrophages are CD80+, M2 macrophages are CD206+. HFD, high fat diet; CD, matched control diet for HFD; PAD, palmitic acid-rich diet; C-PAD, matched control diet for PAD. Data are shown as means ± SEM, n = 4 mice in each group. *p<0.05 compared to CD.

Supplementary Figure S2

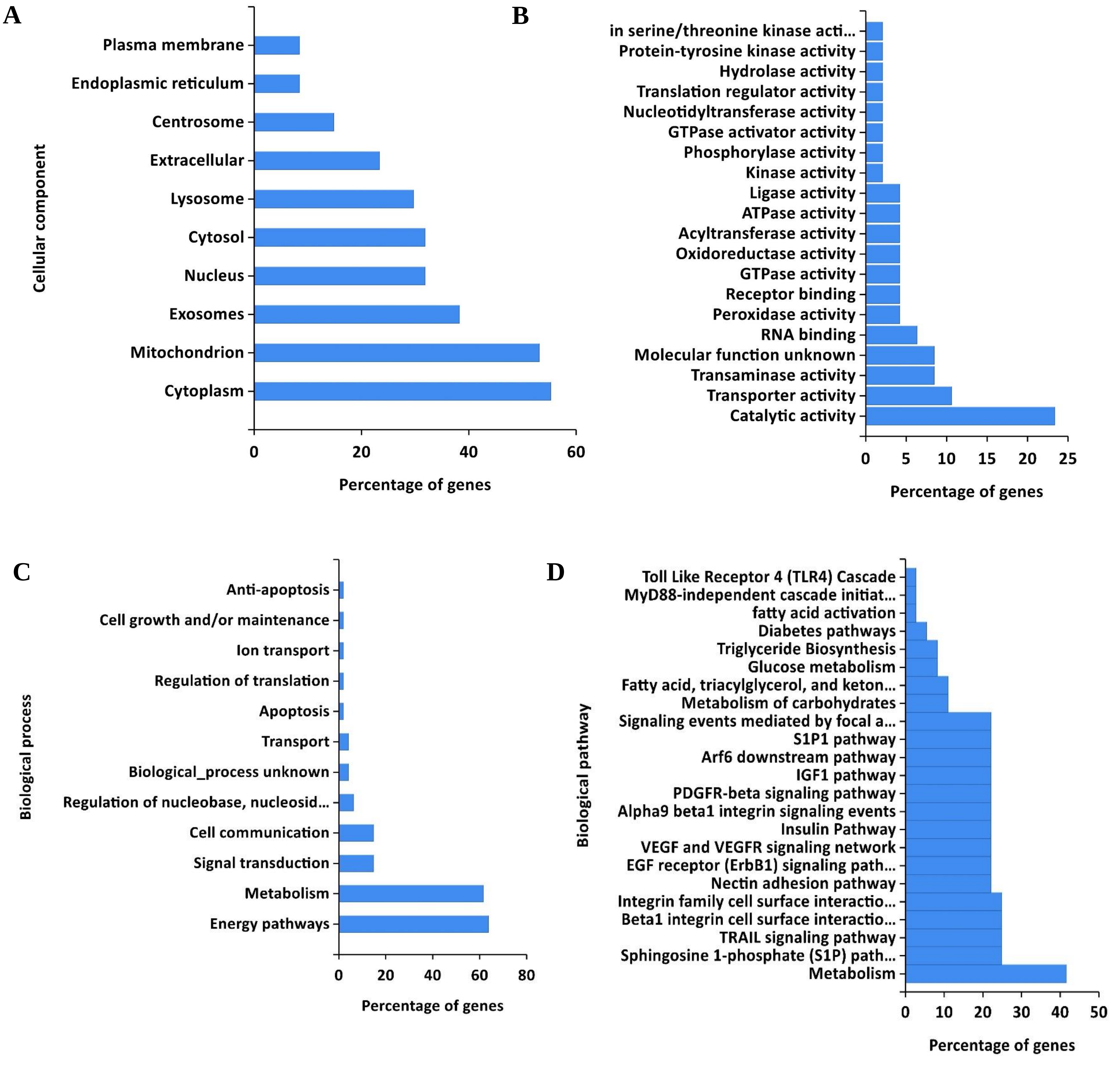

Supplementary Figure S2. GO pathway enrichment analysis on the **(A)** cellular component and **(B)** biological pathway,s and **(C-D)** GO pathway enrichment analysis of the highlighted metabolic enzymes that show significant differences between the wild-type HCT116 or HCT116$^{TLR4\text{-}KO}$ tumor tissues.
