# Supplementary material for: Toll-like receptor 4 is a master regulator for colorectal cancer growth under high-fat diet by programming cancer metabolism
Source: Cell Death Dis. 2021 Aug 12;12(8):791. doi: 10.1038/s41419-021-04076-x (PMC8360949; doi:10.1038/s41419-021-04076-x)
Supplement: Supplementary file 2 — Supplementary Tables [file 41419_2021_4076_MOESM2_ESM.pdf]

Supplementary Table 1

id	HHD_fpkm	HCD_fpkm	HHD_count	HCD_count	Symbol
ENSG00000174125	0	0	0	0	TLR1
ENSG00000137462	0.11	0.06	7	3	TLR2
ENSG00000164342	0.38	0	20	0	TLR3
ENSG00000136869	0.23	0.07	16	4	TLR4
ENSG00000187554	0	0	0	0	TLR5
ENSG00000174130	0	0.03	0	2	TLR6
ENSG00000196664	0	0.05	0	3	TLR7
ENSG00000101916	0	0	0	0	TLR8
ENSG00000239732	0	0	0	0	TLR9
ENSG00000174123	0	0.02	0	1	TLR10

Supplementary Table 1. RNA sequencing analysis of the HHD and HCD tumor tissues. Quantification of the TLR genes in the xenograft tissues. HHD, HFD feeding persisted for 35 days; HCD, HFD feeing for 15 days followed by CD feeding for 20 days. FPKM, fragments per kilobase million; count, total count. n = 4-8 mice in each group.

Supplementary Table 2

Fatty acid	HHD:HCD	p-value	Mass	Retention Time
Myristic acid	up	5.72E-07	228.206	15.210
Oleic acid	up	2.82E-08	282.256	17.744
Palmitic acid	up	7.35E-06	256.240	17.541
Pelargonic acid	up	6.25E-06	316.259	10.450
Pentadecylic acid	up	1.95E-04	302.242	17.619
Stearic acid	up	6.61E-07	284.271	19.615
10-Hydroxy-3,7-dimethyl-2E,6E-decadienoic acid	down	8.92E-06	212.140	8.200
10-tridecynoic acid	up	2.05E-07	210.161	7.020
12-tridecenoic acid	up	5.24E-04	484.374	17.129
12Z,15Z-heneicosadienoic acid	down	3.63E-04	704.585	23.409
12Z-heneicosenoic acid	down	7.62E-04	324.299	19.888
13Z-eicosenoic acid	down	2.79E-05	310.282	19.797
2E,6E,8E,10E-dodecatetraenoic acid	up	1.29E-16	192.115	7.7859
2E,6Z,8Z,12E-hexadecatetraenoic acid	up	6.25E-06	744.524	20.779
2Z,4E-nonadienoic acid	up	5.89E-09	368.216	7.629
3-undecynoic acid	up	4.27E-04	242.152	5.599
4,8,11,14-Eicosatetraynoic acid	up	1.42E-06	592.367	18.187
5,8-heptadecadiynoic acid	up	1.19E-08	322.212	11.762
5-oxo-7-decynoic acid	up	1.54E-04	546.279	10.250
6,9,12,15,18,21-Tetracosahexaynoic acid	up	1.92E-04	404.193	7.624
6E,8E,12E,14E-Hexadecatetraen-10-ynoic acid	up	5.79E-11	488.296	18.641
8,11,14-nonadecatriynoic acid	up	1.69E-04	346.213	14.263
8Z-Decene-4,6-diynoic acid	up	6.54E-04	546.229	19.306
alpha-nonylenic acid	up	0.00147588	216.134	7.8041
beta-ethyl acrylic acid	up	2.21E-08	300.155	8.4722
cis,cis-dodeca-3,6-dienoic acid	up	1.78E-04	196.145	8.383
trans,trans-hepta-2,4,6-trienoic acid	up	1.71E-07	308.120	8.368
trans-beta-octenoic acid	down	2.01E-08	426.305	19.279

Supplementary Table 2. Global lipidomics highlights the fatty acid species that are most significantly affected by the dietary interventions. HHD, HFD feeding persisted for 35 days; HCD, HFD feeing for 15 days followed by CD feeding for 20 days. n = 4-8 mice in each group.

Supplementary Table 3

ng/mg protein	Palmitic acid	Stearic acid	Oleic acid
**CD**	1.0204±0.0281	0.5120±0.0186	4.0387±0.0200
**HD**	3.7802±0.6189[a]	1.5079±0.2044[a,e]	2.5580±0.4097[a,d]
**HHD**	4.6550±0.2315[a]	14.0742±0.3258[c]	0.1180±0.0005[b]
**HCD**	3.0331±0.1777[a,d]	1.2206±0.0292[a,e]	0.0967±0.0046[b]
**Retention time (min)**	17.541	19.615	17.744
**Accurate MS**	256.2402	284.2715	282.2559

Supplementary Table 3. Quantification of the palmitic acid, stearic acid and oleic acid in the tumor tissues of the mouse models under different dietary interventions. Data are shown as means ± SEM. n = 4-8 mice in each group. a< 0.05, b< 0.01, c<0.001 compared to CD; d< 0.05, e<0.001 compared to HHD. CD, match control diet feeding; HD, high fat diet feeding; HHD, HFD feeding persisted for 35 days; HCD, HFD feeing for 15 days followed by CD feeding for 20 days.

Supplementary Table 4

A

Name	Citable Accession	Gene Name	KO-HFD/ HFD	KO-HFD/ HFD	KO-HFD/ KO-CD	KO-HFD/ KO-CD	Mass(KDa)	GRAVY	Isoelectric Point
Isocitrate dehydrogenase	O75874	IDH1	0.501187205	down	0.855066717	--	46.659	-0.39178744	6.53
Apoptosis-inducing factor 1	O95831	AIFM1	0.444631308	down	0.758577585	--	66.9001	-0.268189233	9.04
ATP-dependent RNA helicase	Q92499	DDX1	0.580764413	down	0.772680581	--	82.4317	-0.395540541	6.8
Glyoxalase domain-containing protein 4	Q9HC38	GLOD4	0.636795521	down	1.047129035	--	34.7932	-0.383067093	5.4
Mitochondrial import receptor subunit TOM34	Q15785	TOMM34	0.380189389	down	0.731139123	--	34.559	-0.609385113	9.12
Transforming growth factor-beta-induced protein ig-h3	Q15582	TGFBI	0.597035289	down	1.037528038	--	74.6802	-0.085065886	7.62
Phosphoenolpyruvate carboxykinase	Q16822	PCK2	0.416869402	down	0.920449615	--	70.6984	-0.27546875	7.57
Adipocyte plasma membrane-associated protein	Q9HDC9	APMAP	0.654636085	down	0.972747207	--	46.4799	-0.186298077	5.82
Aldehyde dehydrogenase	P05091	ALDH2	0.349945188	down	0.981747925	--	56.3809	-0.13655706	6.63
Rho GTPase-activating protein 1	Q07960	ARHGAP1	0.492039502	down	0.937561989	--	50.4353	-0.366514806	5.85
NAD(P) transhydrogenase	Q13423	NNT	0.212813899	down	0.809095919	--	113.8946	0.29907919	8.31
Phosphoserine aminotransferase	Q9Y617	PSAT1	0.461317599	down	1.047129035	--	40.4224	0.019459459	7.56
Acetyl-CoA acetyltransferase	Q9BWD1	ACAT2	0.642687678	down	1.116863012	--	41.3505	0.202518892	6.46
Glutamine synthetase	P15104	GLUL	0.224905506	down	0.619441092	down	42.0641	-0.595710456	6.43
Flotillin-1	O75955	FLOT1	0.580764413	down	1.076465011	--	47.355	-0.338407494	7.08
Tyrosine-protein kinase CSK	P41240	CSK	0.630957425	down	0.855066717	--	50.704	-0.268444444	6.62
Glutathione peroxidase 1	P07203	GPX1	0.591561615	down	0.679203629	--	22.0879	-0.069950739	6.15
Carnitine O-acetyltransferase	P43155	CRAT	0.081658237	down	0.586138189	down	70.8571	-0.241533546	8.63
Programmed cell death protein 4	Q53EL6	PDCD4	0.619441092	down	0.990831971	--	51.7348	-0.484008529	5.07
Isocitrate dehydrogenase subunit alpha	P50213	IDH3A	0.510505021	down	1.355188966	--	39.5914	-0.056284153	6.46
Acetyl-coenzyme A synthetase	Q9NR19	ACSS2	0.322106898	down	0.920449615	--	78.5791	-0.27703281	6.02
MAP kinase-activated protein kinase 2	P49137	MAPKAPK2	0.597035289	down	0.672976673	--	45.5674	-0.476	8.87
Mitochondrial Rho GTPase 2	Q8IXI1	RHOT2	0.534564376	down	1.066596031	--	68.1172	-0.102588997	5.55
ATP-binding cassette sub-family D member 1	P33897	ABCD1	0.524807513	down	0.809095919	--	82.9361	-0.055167785	9.09
Beta-hexosaminidase subunit alpha	P06865	HEXA	0.319153786	down	0.963828981	--	60.7023	-0.222306238	5.04
Insulin-like growth factor 2 mRNA-binding protein 1	Q9NZI8	IGF2BP1	0.044463132	down	0.220800504	down	63.4802	-0.470710572	9.26
Acyl-CoA-binding protein	P07108	DBI	0.608134985	down	1.367728949	--	10.0444	-0.897701149	6.12
Long-chain-fatty-acid--CoA ligase 4	O60488	ACSL4	0.229086801	down	1.028015971	--	79.1874	-0.19395218	8.66
Isobutyryl-CoA dehydrogenase	Q9UKU7	ACAD8	0.474242002	down	0.862978518	--	45.0694	-0.050843373	8.12
4-aminobutyrate aminotransferase	P80404	ABAT	0.169044107	down	0.73790431	--	56.4384	-0.2652	8.17
GTP-binding protein Rheb	Q15382	RHEB	0.478630096	down	0.981747925	--	20.4973	-0.094021739	5.65
Acetoacetyl-CoA synthetase	Q86V21	AACS	0.322106898	down	0.779830098	--	75.1436	-0.150297619	5.86
Glutathione peroxidase 2	P18283	GPX2	0.383707315	down	0.765596628	--	21.9538	-0.32	7.64
Galactose-1-phosphate uridylyltransferase	P07902	GALT	0.636795521	down	0.928966403	--	43.3628	-0.565963061	6.49
Pyruvate dehydrogenase kinase isozyme 3	Q15120	PDK3	0.244343102	down	1.08642602	--	46.9385	-0.390394089	8.46
Pyruvate kinase	P14618	PKM	3.630779982	up	1.066596031	--	57.9364	-0.128060264	7.96
ATP-citrate synthase	P53396	ACLY	3.732501984	up	1.08642602	--	120.8383	-0.105267938	6.95
ATP synthase subunit beta	P06576	ATP5B	2.147830009	up	1.30617094	--	56.5594	0.018147448	5.26
2,4-dienoyl-CoA reductase	Q16698	DECR1	2.937649965	up	1.158776999	--	36.0674	-0.019701493	9.35
Acyl-CoA dehydrogenase family member 9	Q9H845	ACAD9	1.819700956	up	1.08642602	--	68.7597	-0.095491143	8.15
ATP synthase subunit d, mitochondrial	O75947	ATP5H	1.995262027	up	1.16949904	--	18.491	-0.554037267	5.21
Fatty acid-binding protein 5	Q01469	FABP5	8.165823936	up	1.047129035	--	15.1644	-0.457777778	6.59
Aspartate aminotransferase	P17174	GOT1	1.584892988	up	1.047129035	--	46.2471	-0.276513317	6.53
ATP synthase subunit alpha	P25705	ATP5F1	2.312064886	up	1.158776999	--	59.7501	-0.066726944	9.16
Insulin-like growth factor 2 mRNA-binding protein 2	Q9Y6M1	IGF2BP2	4.446312904	up	0.801678121	--	66.121	-0.403505843	8.48
Glycogen phosphorylase	P06737	PYGL	1.836537957	up	1.224616051	--	97.1478	-0.32325856	6.71

B

Name	Citable Accession	Gene Name	KO-HFD/ HFD	KO-HFD/ HFD	KO-HFD/ KO-CD	KO-HFD/ KO-CD	Mass(KDa)	GRAVY	Isoelectric Point
Catenin beta-1	P35222	CTNNB1	0.270395786	down	0.972747207	--	85.4959	-0.17503201	5.53

Supplementary Table 4. iTRAQ proteomics analysis reveal the **(A)** metabolic enzymes that were significantly upregulated or downregulated and **(B)** β-catenin level in the tumor tissues. KO-CD, mice inoculated with TLR4-KO HCT116 cells had CD diet; KO-HFD, mice inoculated with TLR4-KO HCT116 cells had HFD diet; HFD, mice inoculated with HCT116 cells had HFD diet.
