# Supplementary material for: Toll-like receptor 4 is a master regulator for colorectal cancer growth under high-fat diet by programming cancer metabolism
Source: Cell Death Dis. 2021 Aug 12;12(8):791. doi: 10.1038/s41419-021-04076-x (PMC8360949; doi:10.1038/s41419-021-04076-x)
Supplement: Supplementary file 4 — Detailed Attribution of Authorship [file 41419_2021_4076_MOESM4_ESM.pdf]

DECLARATION OF CONTRIBUTIONS TO ARTICLE

ADMC

Manuscript Number:	Journal Name:	
CDDIS-21-1300	*Cell Death & Disease*	(the 'Journal')

Proposed Title of the Contribution:

Toll-like receptor 4 is a master regulator for colorectal cancer growth under high-fat diet by programming the cancer metabolism	(the 'Contribution)

Author(s):

Xianjing Hu, Sarwat Fatima, Minting Chen, Keyang Xu, Chunhua Huang, Rui-Hong Gong, Tao Su, Hoi Leong Xavier Wong, Zhaoxiang Bian, Hiu Yee Kwan	(the 'Authors')

For all *CDDis* articles, each person named as an author in the published version must be able to show he or she has contributed substantially to the article.

Authorship credit should be based on 1) substantial contributions to conception and design, acquisition of data, or analysis and interpretation of data; 2) drafting the article or revising it critically for important intellectual content; and 3) final approval of the version to be published. Authors should meet conditions 1, 2 and 3.

Any person who cannot be shown to have made a substantial contribution to the article cannot be listed as an author in the final version. The name of any person who is deemed to have made a minor contribution can, however, appear in the Acknowledgments section of the article.

Please complete the table below to indicate the contributions of all named authors to the manuscript.

Author Full Name:	Specification of Contribution to the Manuscript:
Xianjing Hu	Data curation and formal analysis, writing, funding acquisition
Sarwat Fatima	Data curation and formal analysis, writing
Minting Chen	Data curation and formal analysis, writing
Keyang Xu	Data curation and formal analysis
Chunhua Huang	Data curation and formal analysis
Rui-Hong Gong	Data curation and formal analysis
Tao Su	Review and editing
Hoi Leong Xavier Wong	Review and editing
Zhaoxiang Bian	Conceptualization, review and editing, funding acquisition
Hiu Yee Kwan	Conceptualization,Data curation and formal analysis, writing, review and editing, funding acquisition

Please complete the table below to indicate the contributions of all named authors to the figures.

Figure 1:
Sarwat Fatima, Xianjing Hu, Hiu Yee Kwan

Figure 2:
Sarwat Fatima, Xianjing Hu, Hiu Yee Kwan

Figure 3:
Xianjing Hu, Sarwat Fatima, Minting Chen

Figure 4:
Xianjing Hu, Sarwat Fatima, Minting Chen

Figure 5:
Xianjing Hu, Chunhua Huang, Rui-Hong Gong

Figure 6:
Xianjing Hu, Keyang Xu

Signed for and on behalf of the Author(s):	Print Name:	Date:
[signature]	Kwan Hiu Yee	13th July 2021
